# 임부의 임신스트레스에 영향을 미치는 요인에 관한 단면연구

**DOI:** 10.4069/kjwhn.2022.02.03

**Published:** 2022-03-23

**Authors:** Sook Jung Kang, Min Ji Yang

**Affiliations:** 1 College of Nursing, Ewha Womans University, Seoul, Korea; 1 이화여자대학교 간호대학; 2 Division of Nursing Science, Graduate School, Ewha Womans University, Seoul, Korea; 2 이화여자대학교 일반대학원 간호과학과; 3 Department of Nursing, Jeonbuk National University Hospital, Jeonju, Korea; 3 전북대학교병원 간호부

**Keywords:** Knowledge, Pregnant women, Psychological distress, Social support, 지식, 임부, 스트레스, 사회적 지지

## Abstract

**Purpose:**

The purpose of this study was to investigate the association between maternal knowledge and social support on pregnancy stress among pregnant women in Korea.

**Methods:**

The participants in this study were 148 pregnant women in Korea, recruited from online communities on pregnancy and/or childbirth, from June 2019 to April 2020. The collected data were analyzed using the independent t-test, one-way analysis of variance, Pearson correlation coefficient, and multiple regression.

**Results:**

Participants were at average 18.25±8.28 weeks gestation, 56% were in the second trimester, 31% had one or more health issues in the current pregnancy (e.g., hyperemesis gravidarum), and 76% were first-time mothers. Participants had moderate levels of pregnancy stress (mean, 23.09±7.11 points out of 48) and maternal knowledge (mean, 14.42±4.67 points out of 21), whereas social support was somewhat high (mean 45.88±7.81 points out of 60). Pregnancy stress was weakly negatively correlated with social support (r=–.37, *p*<.001). Main source of pregnancy information (β=–.21, *p*=.011), marital satisfaction (β=–.18, *p*=.036), and social support (β=–.19, *p*=.038) were identified as significant factors affecting pregnancy stress, and these variables had an explanatory power of 22.7% for pregnancy stress.

**Conclusion:**

Based on these findings, nurses should assess pregnancy-related stress during pregnancy and consider main source of pregnancy information and marital satisfaction when providing education or counseling. Moreover, strategies to reduce pregnancy stress through social support are needed to improve the quality of life for pregnant women.

## Introduction

여성에게 임신과 출산은 중요한 과업 중 하나로, 임신한 여성은 주위 사람들로부터 관심을 받으며 행복하고 흥미로운 시간을 가지기도 하지만 입덧, 체중 증가, 피로 등 신체적 변화를 겪게 되고[1] 출산을 통해 직장이나 가족에서 새로운 역할을 부여받아 사회적, 정신적 변화를 겪고 적응하는 취약한 시기를 경험하기도 한다[2]. 2020년 통계청 조사[3]에 따르면 한국의 혼인 건수는 1천 명당 4.2명으로 2011년 이후 계속 감속 추세이며, 2020년 합계출산율은 0.84명으로 최저수준에 도달하였다. 또한, 혼인 연령이 증가함에 따라 2020년 기준 출산 연령은 33.13세로 매년 높아지고 있다. 이러한 저출산 현상과 출산 연령의 증가는 가족 환경의 변화와 여성의 경제활동과 관련되며, 임신, 출산 등 동시에 여러 역할을 책임져야 하는 여성의 심리적 부담감이 높아질 수밖에 없어 임부의 임신스트레스를 파악하는 것은 매우 중요하다[4].

임신스트레스는 임신 전체 기간에 걸쳐 흔하게 일어나며 다양하게 변화하는 연속체로 아기의 건강, 신체적 변화, 임신과 출산, 배우자 및 다른 사람의 관계 등과 관련된 임부의 불안과 우울, 두려움을 의미한다[5]. 임신스트레스 증상은 독립적으로 나타나기도 하지만 우울이나 불안 등의 심리적 증상과 동반되어 나타나기도 한다[6]. 선행연구에서 임신스트레스의 발생률은 측정도구와 임신 시기별로 다양하게 나타나지만, 높게는 78%로 조사된 바 있다[7]. 임신 기간별 임신스트레스의 결과는 임부의 특성과 상황에 따라 다른 양상으로 나타나고 있어 임신 전반기에 걸쳐 임신스트레스를 조사하는 것이 필요하다[8]. 임신스트레스는 유산 및 조기진통을 일으키며 혈압을 증가시키고[9,10], 응급 제왕절개 수술의 증가, 저체중아 출산, 조산, 낮은 APGAR 점수, 태아의 뇌신경 발달 지연 등 산모와 태아 측면에서 임신에 악영향을 미친다[10,11]. 또한 임신스트레스는 아기에 대한 애착이나 양육 태도에 영향을 미치므로 추후 영아 및 유아의 행동, 인지, 정서적 발달과 관련되어 아동의 건강과 삶에 연관성이 있다고 제시되고 있다[12,13]. 임신 기간에 스트레스를 더 많이 경험한 여성은 출산 후 산후우울증, 불안, 산후스트레스에 대한 위험도가 높아지므로 임신스트레스 관리의 중요성은 점차 강조되고 있다[6].

선행연구에서 임신스트레스에 영향을 미치는 요인으로는 나이, 경제적 수준 등의 개인적 측면, 임신 및 출산과 관련된 지식, 부정적인 대처 유형 등의 상황적 측면, 사회적 지지 자원의 부족, 의료진과의 관계 등 환경적 측면으로 보고된 바 있다[14,15]. 특히 모성 관련 지식은 임신과 출산 동안 건강 행위에 필수적이며 인식과 행동에 영향을 미치는 요인으로 알려져 있고, 자가간호 능력을 향상시켜 여성의 건강 및 태아의 성장발달에 긍정적으로 작용한다. 임신, 출산 및 육아에 대한 지식을 습득하는 것은 자신감을 높여 임신스트레스를 줄이므로 매우 중요하다고 볼 수 있다[16]. 하지만 임부들은 지식과 정보를 얻는 방법으로 많이 사용하는 인터넷에서 정확하지 않은 정보나 신념을 얻을 수 있으며, 이러한 정보를 전문가와 상의하지 않고 따르면 매우 위험하거나 혼란스러움을 일으켜 불필요한 걱정을 하게 된다[17]. 따라서 임부들의 올바른 모성 관련 지식이 임신스트레스에 어떠한 영향을 미치는지 규명할 필요가 있다.

사회적 지지는 개인의 가치나 심리적 안녕감을 제공할 뿐만 아니라 자원에 접근할 수 있도록 도와주고 임신 중 부정적인 심리상태를 완화시켜 준다[18]. 또한 임신 중 사회적 지지가 부족하여 적절한 지원을 받지 못하면 스트레스에 영향을 미치게 되는데, 배우자와의 관계가 만족스럽지 않은 여성은 만족하는 여성에 비해 임신스트레스를 경험할 가능성이 약 4배 높은 것으로 나타났다[19]. 때문에 삶의 전환기라 할 수 있는 임신 기간 동안 임부를 지지하는 데 있어 배우자와 가족 구성원의 역할은 매우 중요하다[18]. 임신은 임부 혼자서 겪어야 할 일이 아니라 배우자와 가족, 친구 모두 함께 해결해야 할 과업이므로, 사회적 지지가 임신스트레스에 미치는 영향을 확인하는 것은 매우 의미있다고 하겠다.

임부의 임신스트레스는 모성 관련 지식, 사회적 지지 뿐 아니라 나이, 경제적 수준, 결혼상태, 동거가족, 직업 등 일반적 특성과 임신 주수, 계획임신 여부, 자녀 수, 임신 중 산과 문제 등 임신 관련 특성에 따라서 다르게 나타났다[14,15]. 이러한 다양한 인구학적 요소들이 임신스트레스에 어떠한 관련성이 있는지 확인할 필요가 있다.

최근까지 임신스트레스와 관련한 연구는 임신 및 출산 후 우울, 불안 등 심리적인 요소와의 관계나 원인을 파악하는 연구[20,21], 임신스트레스가 출산 결과에 영향을 미치는 요인임을 확인한 연구[10,11], 임신스트레스를 감소시키기 위한 중재 프로그램을 개발한 연구[22], 임신 전과 임신 중에 측정한 임신스트레스에 관한 문헌고찰 연구[23] 등 국내외에서 다양하게 이루어지고 있다. 하지만 모성 관련 지식, 사회적 지지 및 임신스트레스를 동시에 확인한 연구는 찾아보기 힘들고, 특히 임부 대상의 모성 관련 지식에 대한 연구는 매우 부족하다. 또한 임신 중 우울, 불안의 관련 요인을 확인한 연구는 많았으나 임신스트레스의 관련 요인을 확인한 연구는 미비하였으며, 부정적 심리 요소와의 관련성 등 단편적 연구에 그쳐 다양한 영역을 통합적으로 확인하는 연구가 필요하다.

따라서 본 연구는 모성 관련 지식과 사회적 지지의 부족이 임신스트레스에 위험요인으로 작용한다는 점을 인식하고 이를 통해 임부의 임신스트레스를 관리하는 포괄적인 전략을 수립하며 중재 프로그램의 기초 자료로 활용하고자 시도하였다.

본 연구의 목적은 임부의 모성 관련 지식과 사회적 지지가 임신스트레스에 미치는 영향을 파악하고자 함이며, 구체적인 목적은 다음과 같다.

(1) 임부의 임신스트레스, 모성 관련 지식 및 사회적 지지 정도를 파악한다.

(2) 임부의 특성에 따른 임신스트레스의 차이를 파악한다.

(3) 임부의 임신스트레스, 모성 관련 지식, 사회적 지지의 관계를 파악한다.

(4) 임부의 임신스트레스에 영향을 미치는 요인을 파악한다.

## Methods

Ethics statement: Written informed consent was exempted by the Institutional Review Board of Ewha Womans University (EWHA-201905-0020) due to the nature of the online survey. Explanations were provided that participation in the online questionnaire is regarded as consent and all data were anonymously treated.

### 연구 설계

본 연구는 모성 관련 지식과 사회적 지지가 임신스트레스에 영향을 미치는 요인임을 규명하기 위한 상관성 조사연구이다. 본 연구는 STROBE 보고지침(https://www.strobe-statement.org)에 따라 기술하였다.

### 연구 대상 및 표집 방법

본 연구는 임부를 대상으로 시행한 설문조사 연구로 임부들의 사이버 모임인 인터넷 카페에 대상자 모집 문건을 게시한 후 자발적으로 동의한 대상자만 참여하였다. 대상자의 구체적인 선정기준은 4–5주경부터 임신낭(gestational sac)을 확인할 수 있기 때문에[24] 전문의의 진단으로 임신이 확인된 4주 이상의 출산 전 여성, 만 18세 이상으로 한국어를 읽고 이해할 수 있고 의사소통이 가능한 여성이며, 제외기준은 지남력의 장애가 있으며, 임신 전부터 정신과 질환을 가진 여성으로 하였다. 연구 대상자 수는 Cohen [25]의 Power analysis 공식에 근거한 G*Power 3.1.9.2 프로그램을 이용하였고, 다중회귀분석에서 필요한 최소한의 표본 크기를 계산한 결과 임신스트레스 영향요인을 확인한 선행연구[26]를 근거로 유의수준 .05, 중간효과 크기 .18, 검정력 0.8로 가정하였다. 예측변수는 일반적 특성 10개, 임신 관련 특성 11개, 독립변수(모성 관련 지식, 사회적 지지) 2개를 합하여 총 23개를 설정하였다[14]. 그 결과 최소 표본 수는 142명으로 산출되었으나, 탈락률을 10%로 감안하여 최종 160명을 목표로 하고 150명을 모집하였다. 설문지 중 응답이 불완전하거나 불성실한 설문지 2부를 제외한 148부를 최종 자료 분석에 이용하였다.

### 연구 도구

본 연구에서 사용한 도구는 모성 관련 지식, 사회적 지지, 임신스트레스 도구이다. 이들 측정도구를 도구 개발자와 번안자의 승인을 받고 사용하였다.

#### 임신스트레스

본 연구에서 임신스트레스는 Pop 등[27]이 개발한 Tilburg Pregnancy Distress Scale (TPDS)를 개발자로부터 허락을 구한 후 영어와 한국어가 능통한 영문학 전공자가 도구 번역 가이드라인 과정을[28] 참고하여 번역과 역번역의 과정을 거쳐 진행하였다. 번역된 임신스트레스 도구는 산과 경력 5년 이상의 간호사 3인, 조산사 1인, 산부인과 의사 1인으로 구성된 전문가 집단에게 내용 타당도에 대한 자문을 구하였다. 내용 타당도 지수(Content Validity Index Item [I-CVI])는 .60–1.00으로 평균 .95이었고, 총 16문항 중 한 가지 문항(14번 문항: “임신과 관련된 건강문제들이 출산 이후에도 계속될까 염려된다”)이 .60이었으나 전문가 집단과 상의한 결과 중요한 문항으로 판단되어 포함하기로 하였으며 S-CVI (Scale-level Content Validity Index [S-CVI]/Ave)는 .925이었다. 현직 국어 교사 1인에게 문항의 적절성을 검증받은 후 설문지를 완성하였다. 임신스트레스는 원 도구대로 총 16문항으로, 부정적 정서(negative affect), 배우자 참여(partner involvement)의 하위 영역으로 구성되어 있다. 각 문항은 4점 Likert 척도(‘매우 자주 그렇다’ 0, ‘거의 그렇지 않다’ 3)로 합산점수가 최저 0점에서 최고 48점이며, 점수가 높을수록 임신스트레스가 높음을 의미한다. TPDS는 절단점수(cut-off value)가 있는 도구로 총 점수가 17점 초과이거나 부정적 정서 영역은 12점 초과, 배우자 참여 영역은 7점 초과일 때 유의미한 것으로 간주한다. 도구 개발 당시 신뢰도는 Cronbach’s α=.78이었고, 본 연구에서는 Cronbach’s α=.84이었다.

#### 모성 관련 지식

본 연구에서 모성 관련 지식은 Kim [29]이 개발한 도구를 시대적 상황에 맞도록 연구자가 수정 및 보완하였다. 예를 들면 우유를 모유나 분유로 수정하였고, 기초체온법, 배란일 계산법, 경구피임약 부작용 등의 문항을 삭제하였으며, 임신과 출산 시 적용되는 국가정책 및 혜택, 산전검사 방법 및 시기, 출산 후 변화 및 관리방법 등을 추가하였다. 산과 경력 5년 이상의 간호사 3인, 조산사 1인, 모성간호학 전공자 1인으로 구성된 전문가 집단에게 내용 타당도를 확인받은 후 사용하였다. I-CVI는 .80–1.00으로 평균 .97이었다. 모성 관련 지식은 총 21문항으로 이루어져 있으며, 각 문항은 ‘예’ 1점, ‘아니요’ 0점으로 측정된다. 합산점수 범위는 최저 0점에서 최고 21점으로, 점수가 높을수록 지식 정도가 높음을 의미한다. 도구 개발 당시 신뢰도는 Cronbach’s α=.82이었고, 본 연구에서는 Kuder-Richardson-20 (KR-20)=.88이었다.

#### 사회적 지지

본 연구에서 임부의 사회적 지지는 Zimet 등[30]이 개발한 Multidimensional Scale of Perceived Social Support (MSPSS)의 한국어판 도구를 원 도구 개발자로부터 사용 승인을 받아 사용하였다. MSPSS는 총 12문항으로 구성되어 있으며 가족, 친구, 의미 있는 타인으로부터 지각된 지지를 측정한 도구이다. 각 문항들은 5점 척도이며(‘매우 그렇지 않다’ 1, ‘매우 그렇다’ 5), 합산점수가 최저 12점에서 최고 60점으로 점수가 높을수록 사회적 지지가 높음을 의미한다. 도구 개발 당시 신뢰도는 Cronbach’s α=.83이었고, 본 연구에서는 Cronbach’s α=.91이었다.

#### 일반적 특성 및 임신 관련 특성

일반적 특성은 임부의 나이, 교육 수준, 종교, 직업, 결혼 만족 등 10개의 항목으로 이루어졌으며. 임신 관련 특성은 계획임신 여부, 임신 방법, 임신 시 주된 정보 출처(미디어 또는 가족, 친구) 등 11개 항목으로 연구자가 문헌을 바탕으로 구성하였다.

### 자료 수집

본 연구는 2019년 6월 1일부터 2020년 4월 30일까지 임신 및 출산과 관련 인터넷 카페를 통한 온라인 설문조사로 자료를 수집하였다. 설문조사는 인터넷 카페 운영자에게 협조를 구하였고, 인터넷 카페 게시판에 설문조사 링크를 게시한 후 링크된 주소로 접속할 경우 온라인 설문지에 연결하여 작성하는 방법으로 진행하였다. 온라인 설문은 연구의 목적, 취지, 절차, 연구 참여자의 권리, 비밀 보장을 설명하고 자발적 동의를 받아 진행했으며, 연구과정의 모든 자료 및 결과는 연구 목적 이외 사용하지 않을 것임을 설명하였다. 설문지를 작성할 때 문항으로 불편감을 느낄 경우 휴식을 취한 후 설문을 작성하도록 안내했으며, 휴식 후에도 불편감이 있으면 중도에 설문 작성을 중단해도 됨을 설명하였다. 산과적 특성과 관련하여 민감하게 느낄 수 있는 질문에는 객관식 선택지로 답변할 수 있도록 설문지를 구성하였고, 연구자에게 연락을 취하고자 할 때 익명으로 연락할 수 있도록 하였다. 설문지 작성에 소요된 시간은 10−15분 정도였고, 설문을 완료한 후 소정의 선물을 증정하였다.

### 자료 분석 방법

자료 분석은 IBM SPSS for Windows ver. 25.0 (IBM Corp., Armonk, NY, USA)을 사용하였고, 통계적 유의성은 *p*<.05로 하였으며 구체적 내용은 다음과 같다.

(1) 임부의 일반적 특성과 임신 관련 특성, 모성 관련 지식, 사회적 지지, 임신스트레스에 관한 실수와 백분율, 평균과 표준편차 등 기술통계로 분석하였다.

(2) 임부의 모성 관련 지식, 사회적 지지, 임신스트레스 정도는 평균과 표준편차 등 기술통계로 분석하였고 임부의 특성에 따른 임신스트레스 차이는 독립 t-검정(independent t-test) 및 일원분산분석(one-way analysis of variance), 사후 분석은 Scheffé test로 분석하였다.

(3) 임부의 모성 관련 지식, 사회적 지지, 임신스트레스 간의 상관관계는 피어슨 상관계수(Pearson correlation coefficients)로 분석하였다.

(4) 임부의 임신스트레스에 영향을 미치는 요인을 파악하기 위해 입력(entered)방법에 의한 다중회귀분석(multiple regression)으로 분석하였다.

## Results

### 대상자의 특성 및 일반적 특성에 따른 임신스트레스 차이

연구 대상자의 일반적 특성을 살펴보면, 대상자의 평균 나이는 평균 32.48±3.79세로 35세 이상이 46명(31.1%)이었고, 최저 22세에서 최고 42세의 범위를 나타냈다. 임부의 교육 수준은 대졸 이상이 110명(74.3%), 종교가 있는 경우가 81명(54.7%), 직업이 있는 경우가 85명(57.4%), 월평균 수입은 400만원 이상인 경우가 76명(51.3%)으로 가장 많았다. 남편과의 관계는 원만한 경우가 133명(89.9%), 시댁과의 관계는 원만한 경우가 95명(64.2%), 친정 부모와의 관계는 원만한 경우가 129명(87.2%), 결혼 만족은 만족하는 경우가 123명(83.1%)이었다.

대상자의 임신 관련 특성은 계획임신인 경우가 121명(81.8%)이었고 임신 방법은 자연임신이 124명(83.8%)이었다. 임신유형은 단태 임신인 경우가 143명(96.6%)이었고, 유산 경험이 없는 경우가 124명(83.8%)이었다. 대상자의 임신 주수는 평균 18.25±8.28주로 56.1%가 임신 2기였고, 자녀가 없는 경우가 113명(76.4%)이었으며 산전 교육 경험이 없는 임부가 88명(59.5%)이었다. 임신 관련 정보 출처는 대중매체가 122명(82.4%)으로 가장 많았고, 이전 임신에서 산과적 문제가 없는 경우가 122명(82.4%), 현재 임신 동안 산과적 문제가 없는 경우가 102명(68.9%), 임신 전 건강 문제가 없는 경우가 140명(94.6%)으로 나타났다. 임신 동안 산과적 문제로는 임신 오조증이 29명(56.9%)으로 가장 많았다.

임신스트레스는 직업(t=–2.42, *p*=.017), 시댁과의 관계(t=–2.17, *p*=.032), 결혼 만족(t=–3.79, *p*<.001), 계획임신(t=–2.40, *p*=.022), 임신 주수(F=4.18, *p*=.017), 임신 관련 정보 출처(t=–3.06, *p*=.005)에 따라 유의한 차이가 있는 것으로 나타났다. 사후 분석 결과 임신 1기와 2기 군이 임신 3기 군보다 임신스트레스가 높은 것으로 나타났다(Table 1).

### 임부의 임신스트레스, 모성 관련 지식 및 사회적 지지 정도

본 연구에서 대상자의 임신스트레스는 평균 23.09±7.11점으로 중간 정도 수준이었다. 임신스트레스를 가진 임부는 117명(79.1%)으로 조사되었다. 임신스트레스의 하위영역인 부정적 정서 영역은 평균 19.94±6.10점이었고, 배우자 참여 영역은 평균 3.16±2.90점이었다. 모성 관련 지식은 평균 14.42±4.67점으로 중간 수준이었고, 사회적 지지는 평균 45.88±7.81점으로 약간 높은 수준이었다(Table 2).

### 임신스트레스, 모성 관련 지식, 사회적 지지와의 관계

본 연구에서 대상자의 임신스트레스는 사회적 지지와 약한 음의 상관관계(r=–.37, *p*<.001)가 있는 것으로 나타났고, 모성 관련 지식은 사회적 지지와 약한 양의 상관관계(r=.28, *p*=.001)가 있는 것으로 나타났다(Table 3).

### 임신스트레스에 미치는 영향요인

임신스트레스에 영향을 미치는 요인을 확인하기 위해 대상자 특성 중 임신스트레스에 유의한 차이가 있었던 임부의 직업, 시댁과의 관계, 결혼 만족, 계획임신, 임신 주수, 임신 관련 정보 출처는 더미 변수 처리 과정을 거쳤다. 임신스트레스 관련 요인인 모성 관련 지식, 사회적 지지와 함께 독립변수로 투입하여 다중회귀분석을 실시하였다.

회귀분석에서 기본적으로 검토해야 할 사항으로 선형성 및 등분산성을 검정하기 위해 산점도를 확인한 결과, 잔차의 분포가 0을 중심으로 균등하게 흩어져 있으므로 가정을 충족하였다. 또한 회귀 표준화 잔차의 정규 P-P 도표를 이용하여 오차의 정규분포를 확인한 결과 45° 직선에 근접해 있으므로 오차는 정규분포를 이룬다고 할 수 있다. 오차의 독립성을 검증하기 위해 Durbin-Watson 통계량을 확인한 결과, 2.137로 2에 가까워 모형의 오차항 간에 자기 상관성은 없는 것으로 나타났다. 독립변수 간 다중공선성은 공차한계와 variance inflation factor (VIF) 지수를 이용하였다. 독립변수 간 VIF 지수는 1.07–1.54로 10 미만이었으며, 공차 한계는 0.65–0.94로 0.1 이상으로 나타나 다중공선성에 문제가 없는 것으로 나타났다.

다중회귀분석 결과 결혼 만족((β=–.18, *p*=.036), 임신 관련 정보 출처(β=–.21, *p*=.011), 사회적 지지(β=–.19, *p*=.038)가 임신스트레스에 영향을 미치는 요인으로 나타났으며 회귀모형은 통계적으로 유의한 것으로 나타났다(F=5.80, *p*<.001). 이러한 변수가 임부의 임신스트레스를 설명하는 설명력은 22.7%였다. 즉, 임신 관련 정보를 친구와 가족으로부터 얻는 임부, 결혼생활에 만족하는 임부, 그리고 사회적 지지가 높은 임부일 경우 임신스트레스가 낮은 것으로 나타났다. 임부의 임신스트레스에 영향을 미치는 변인에 대한 상대적 중요도에서는 임신 관련 정보 출처(β=–.21)가 가장 높았고, 사회적 지지(β=–.19), 결혼 만족(β=–.18) 순으로 나타났다(Table 4).

## Discussion

본 연구 대상자의 임신스트레스 점수는 총 48점 만점에 평균 23.09±7.11점이었다. 동일한 도구인 TPDS를 이용한 터키 임부를 대상으로 한 연구[31,32]에서는 평균 15.72±9.31점, 14.81±7.18점이었고, 네덜란드 임부 396명을 대상으로 임신스트레스를 조사한 연구[33]에서는 평균 12.54±6.6점이었으며, 네덜란드 임부 1,739명을 대상으로 임신 12주, 22주, 32주에 임신스트레스를 측정한 연구[34]에서는 각각 평균 10.7점, 10.3점, 10.9점으로 나타났다. 본 연구 대상자의 임신스트레스는 선행연구의 임신스트레스보다 2배 가량 높은 점수로, 임신스트레스가 매우 높음을 의미한다. 국내에서 TPDS 도구를 사용한 선행연구가 없어 직접 비교가 힘들고 신중한 논의가 필요하지만, 본 연구에서 임신스트레스를 가진 임부는 총점 17점이 초과된 임부로 79.1%를 차지하였는데 선행연구[33]에서는 19.9%를 차지하는 것으로 나타났다. 본 연구의 대상자는 임신 합병증을 가진 임부와 일반 임부 모두를 포함한 데 반해, 선행연구[31-33]는 임신 합병증을 가진 대상자는 제외하고 일반 임부만을 대상자로 하였기 때문으로 여겨진다. 또한 본 연구에서 임신 주수는 임신 1기(32.4%)와 2기(56.1%)보다 3기(11.5%)가 다소 적었으나 선행연구[31-33]에서는 임신 1기가 적고 임신 2기, 3기가 90% 이상을 차지하고 있어 임신 주수별 분포가 다르기 때문으로 생각된다. 조기 진통 등 임신 합병증을 가진 임부가 정상적인 임부보다 임신스트레스가 높다는 선행연구[20]의 결과와 비교하여 볼 때, 임신 합병증을 가진 여성의 정신건강 관리가 매우 필요하다 하겠다. 또한 한국 임부가 임신과 출산에 대한 부담이 크고 전통적으로 부정적인 감정의 억제와 정서적 통제가 스트레스를 악화시킬 수 있을 것으로 보여 한국 임부와 국외 임부의 임신스트레스를 비교하는 후속 연구가 필요할 것으로 생각된다.

또한, 임신 기간별로 임신스트레스를 확인한 결과 임신 1기, 2기가 3기보다 높은 것으로 조사되었다. 임신스트레스를 조사한 선행연구들의 결과는 모두 일치하지 않고 상반되는 결과를 보이고 있는데, 많은 연구에서 신체 변화 및 환경의 변화를 겪는 임신 초기와 출산을 앞둔 말기에 임신스트레스가 높음을 알 수 있었지만[32,34], 중기에서 스트레스가 높은 선행연구[8]도 있어 반복 연구가 필요하다. 또한 임부가 인지한 우울, 불안, 스트레스를 조사한 선행연구[21]에서 임신스트레스의 발생률은 16%로, 불안 31%, 우울감 22%에 이어 높은 비율을 차지하고 있는 것으로 나타나 임신스트레스 관리의 중요성을 시사해 준다. 따라서 임신 초기, 중기, 말기에 임신스트레스를 사정하고 선별해야 하며 임부의 정신건강 서비스 개선 및 교육을 제공하기 위한 전략이 필요하다.

본 연구의 임신스트레스 영역별로 살펴보면, 부정적 정서 영역은 평균 19.94±6.10점이었고 배우자 참여 영역은 평균 3.16±2.90점이었다. 터키 연구[31,32]에서는 부정적 정서 영역이 평균 10.31±7.59점과 10.15±6.46점, 배우자 참여 영역이 평균 5.42±3.48점과 4.66±3.16점이었고, 네덜란드 연구[34]에서는 12주, 22주, 32주에 부정적 정서 영역이 각각 평균 6.49점, 6.01점, 6.47점, 배우자 참여 영역은 각각 평균 4.22점, 4.33점, 4.77점으로, 본 연구의 부정적 정서 영역은 선행연구들보다 매우 높은 점수로 나타났으나 배우자 참여 영역은 선행연구보다 점수가 낮았다. 이러한 결과는 임신스트레스 중 배우자와의 관계보다는 임신, 출산, 산후 관리에 대한 불안이나 걱정이 임신스트레스의 비중에서 크게 차지한다고 볼 수 있다. 이는 여성들의 심리적 고통을 감소시키기 위해 임신과 출산 관리를 제공하는 건강 전문가들이 임신스트레스 프로그램의 개발 및 중재 시에 심리적 측면에 많은 주의를 기울여야 한다는 의견을 뒷받침해준다.

본 연구에서 대상자의 특성 중 임신 관련 정보 출처가 임신스트레스에 가장 많은 영향을 미치는 요인으로 확인되었고, 친구와 가족으로부터 정보를 획득하는 군이 임신스트레스가 낮은 것으로 나타났다. 이러한 결과는 임신 과정 동안 지각된 불안과 걱정을 해결하기 위해 임신 및 출산 정보를 습득하는 것이 임신을 유지하고 적응하는 데 도움이 되는 과정이라고 본 연구[17]를 지지해 주는 결과라 할 수 있다. 배우자 및 가족의 역할은 임신과 출산 전 기간에 걸쳐 중요하기 때문에 간호를 제공할 때 가족, 특히 배우자의 참여를 격려하는 것이 효율적이라고 생각되며, 이를 활성화할 수 있는 구체적인 방안과 적극적인 관심이 필요해 보인다. 또한 가족으로부터 정보를 획득하는 것이 임신스트레스를 감소시킬 수 있기 때문에, 가족 중 누구로부터 정보를 얻는지, 그 정보는 어떤 도움이 되는지 등을 확인하는 것이 필요할 것이다. 더불어 본 연구에서는 임신 관련 정보 출처로 의료진을 포함하지 않았는데, 의료진으로부터 얻은 임신 관련 정보가 임신스트레스에 어떻게 영향을 미치는지 등의 후속 연구 검증이 뒷받침되어야 할 것이다.

최근 정보화로 인해 임부가 임신, 출산, 산후 과정 동안 정보 획득을 위해 인터넷을 많이 이용하는데 이러한 정보는 부정확하거나 파편적 지식으로 이루어진 경우가 많다[17]. 때문에 이로 인한 혼란이 불안과 걱정을 야기하여 더 큰 정신건강 문제의 원인이 될 수 있다. 따라서 의료 전문가가 제공하며 신뢰할 수 있는 임신, 출산, 산후 관련 정보를 제공하는 인터넷 기반 시스템을 구축한다면 매우 유용할 것이다.

본 연구에서 임신스트레스 영향요인 중 두 번째는 대상자의 특성 중 결혼 만족으로, 결혼에 만족하는 군이 임신스트레스가 낮은 것으로 나타났다. 결혼 만족이 높을수록 배우자의 지지가 높고, 산후 우울을 예측하는 요인 중 결혼 만족이 중요한 요인임이 보고된 바 있어[26,35], 결혼생활의 만족도는 임부 삶의 질에 중요한 요소임을 알 수 있다. 산전 관리 프로그램 개발 시 결혼 만족 정도를 사정하고 부부가 함께 할 수 있는 스트레스 관리 감소 프로그램 중재를 고려해 볼 필요가 있다. 본 연구에서는 결혼 만족을 단일문항으로 조사하였는데, 결혼 만족도 도구를 이용하여 다양한 측면에서 심층적으로 임신스트레스와의 관계 및 원인을 조사하는 반복 연구도 필요할 것으로 보인다.

마지막으로 본 연구에서 사회적 지지는 임신스트레스에 영향을 미치는 요인으로 확인되었는데, 대상자의 사회적 지지가 높을수록 임신스트레스는 낮은 것으로 나타났다. 연구 대상자의 사회적 지지는 60점 만점 중 평균 45.88±7.81점으로 국내에서 같은 도구를 이용하여 임부 129명의 사회적 지지를 측정한 연구[36]의 평균 48.57±6.85점, 185명의 임부에게 사회적 지지를 측정한 국외 연구[37]의 평균 43.92±8.5점과 유사한 결과를 보였다. 사회적 지지는 스트레스가 많은 임신 동안 배우자, 가족, 건강 전문가 등을 통해 신체적, 심리적인 안녕을 임부에게 제공하는 역할을 하기 때문에 체계적 지원이 필요하다. 사회적 지지 체계를 강화하는 개별 상담 및 간호중재가 이루어져야 하며, 이는 특히 임신 합병증이나 산후 합병증을 가진 임부에게 더욱 필요할 것으로 생각된다. 또한 지지 체계인 배우자와 가족의 태도가 중요하므로 임부의 문제가 아닌 가족, 더 나아가 지역사회의 공동 문제로 인식하는 문화 및 지속적인 제도가 필요할 것으로 보인다.

본 연구에서 모성 관련 지식은 임신스트레스에 영향을 미치는 요인으로 확인되지 않아, 모성 관련 지식은 임부의 불안을 완화하고 궁금증을 해결해 준다고 제시한 연구[16,17]와 차이가 있었다. 본 연구에 적용된 모성 관련 지식 도구는 임신 동안 알고 있는 기존 지식에 대해 “예”와 “아니오”로 자가 체크하는 단순 응답으로 이루어져 있어, 전문적인 모성 관련 지식을 반영하지 못 했을 수 있다. 모성 관련 지식을 전문적이고 정확하게 측정하여 임신스트레스에 어떤 영향을 주는지에 대해 후속 연구가 필요하다. 특히 본 연구에서 모성 관련 지식이 초임부, 산전 교육 경험이 없는 임부의 경우 점수가 낮았기 때문에 모성 관련 지식을 향상할 수 있는 산전 교육 프로그램에 대한 고려가 필요하다.

본 연구는 임부의 모성 관련 지식, 사회적 지지, 임신스트레스 정도를 파악하고 임신스트레스에 영향을 미치는 요인을 확인하여 임신스트레스를 감소하기 위한 간호 중재 프로그램의 자료로 제공하고 활용하고자 수행하였다. 연구 대상자인 임부의 임신스트레스는 임신 관련 정보 출처를 친구와 가족으로부터 얻은 군, 결혼에 만족하는 군, 사회적 지지가 높은 군일수록 낮은 것으로 나타났다. 본 연구는 대상자가 일반적 건강 문제 및 산과적 문제가 있는 임부를 포함하고 있고 임신 주수의 분류 없이 확인했으며 자료수집이 10개월 동안 진행되어 변화하는 임신 지원정책을 반영하지 못했다는 제한점이 있다. 본 연구에서 사용한 TPDS 도구는 네덜란드에서 제작한 도구로, 본 연구에서는 도구의 내용 타당도를 확인하여 진행하였고 모성 관련 지식 도구 또한 내용 타당도를 확인하였다. 추후 도구의 타당도 확인을 위해 구성과 준거 타당도를 확인하는 것이 필요하다. 이러한 점을 고려하여 임부의 임신스트레스에 영향을 미치는 요인을 파악하는 추가 연구가 필요하다고 생각한다.

그러나 본 연구는 임부의 외적 요인인 모성 관련 지식 및 사회적 지지와 내적 요인인 임신스트레스를 포괄적으로 확인하였고, 관계적 요소인 사회적 지지가 임신스트레스에 영향을 미치는 요인임을 확인한 데 의의가 있다고 볼 수 있다. 본 연구 결과를 토대로 임부의 임신스트레스를 감소시키고 나아가 삶의 질을 향상시킬 수 있을 것으로 기대한다.

이상의 결과를 바탕으로, 임부의 임신스트레스에 영향을 미치는 요인을 중심으로 한 간호 중재 프로그램을 개발, 적용하여 효과를 검증하는 연구가 필요하다. 또한 종단연구를 통해 임부의 초기, 중기, 말기 단계별로 임신스트레스의 변화 양상을 확인하고 임신스트레스에 미치는 요인을 파악하는 후속 연구를 제언한다. 더불어 임신한 여성의 내적 요인과 더불어 외적 요인과 함께 다양한 영역에서 임신스트레스에 미치는 요인을 확인할 필요가 있다.

## Figures and Tables

**Table 1.** Characteristics of pregnant women and differences in pregnancy stress according to characteristics (N=148)

Variable	Categories	n (%) or mean±SD	Pregnancy stress	
			Mean±SD	t or F (*p*)
*General characteristics *				
Age (year)	Range, 22–42		32.48±3.79	
	<35	102 (68.9)	23.47±7.15	0.96 (.340)
	≥35	46 (31.1)	22.26±7.03	
Level of education	<High school	13 (8.8)	24.46±3.69	0.33 (.720)
	College	25 (16.9)	23.44±7.48	
	≥University	110 (74.3)	22.85±7.36	
Religion	Yes	81 (54.7)	22.19±7.59	–1.72 (.087)
	No	67 (45.3)	24.19±6.38	
Occupation	Yes	85 (57.4)	21.89±7.70	–2.42 (.017)
	No	63 (42.6)	24.71±5.93	
Monthly family income (KRW)	<3 million	14 (9.5)	24.00±6.78	0.20 (.822)
	3–4 million	58 (39.2)	23.22±8.53	
	≥4 million	76 (51.3)	22.83±5.97	
Marital status	Married	143 (96.6)	22.99±7.05	–0.99 (.322)
	Unmarried	5 (3.4)	26.20±9.26	
Relationship with husband	Amicable	133 (89.9)	22.81±6.81	–1.13 (.278)
	Average to not amicable	15 (10.1)	25.60±9.33	
Relationship with in-laws	Amicable	95 (64.2)	22.16±6.71	–2.17 (.032)
	Average to not amicable	53 (35.8)	24.77±7.56	
Relationship with parents	Amicable	129 (87.2)	22.91±7.08	–0.80 (.425)
	Average to not amicable	19 (12.8)	24.32±7.42	
Marital satisfaction	Satisfied	123 (83.1)	22.14±6.57	–3.79 (<.001)
	Moderate to dissatisfied	25 (16.9)	27.80±7.93	
*Pregnancy-related characteristics*				
Planned pregnancy	Yes	121 (81.8)	22.33±6.56	–2.40 (.022)
	No	27 (18.2)	26.52±8.51	
Method of pregnancy	Spontaneous	124 (83.8)	23.32±7.37	0.88 (.377)
	Assisted reproductive technologies	24 (16.2)	21.92±5.57	
Type of pregnancy	Singleton	143 (96.6)	23.05±7.22	–0.42 (.678)
	Multiple	5 (3.4)	24.40±2.61	
History of abortion	Yes	24 (16.2)	22.67±7.25	–0.32 (.749)
	No	124 (83.8)	23.18±7.11	
Gestational period (week)	Range, 4–40		18.25±8.28	
	1st trimester^a^	48 (32.4)	23.23±7.16	4.18 (.017)
	2nd trimester^b^	83 (56.1)	23.94±6.69	a,b>c^‡^
	3rd trimester^c^	17 (11.5)	18.59±7.74	
Number of children	≥1	35 (23.6)	23.46±7.80	0.34 (.731)
	0	113 (76.4)	22.98±6.92	
Experience of prenatal education	Yes	60 (40.5)	24.20±7.32	1.57 (.119)
	No	88 (59.5)	22.34±6.91	
Main source of pregnancy information	Mass media (internet, pamphlet)	122 (82.4)	24.08±6.28	–3.06 (.005)
	Family & friends	26 (17.6)	18.46±8.92	
Obstetrical complications in previous pregnancy	Yes	26 (17.6)	22.54±7.68	–0.44 (.662)
	No	122 (82.4)	23.21±7.02	
Obstetrical complications during current pregnancy	Yes^†^	46 (31.1)	23.41±7.35	0.37 (.716)
	Hyperemesis gravidarum	29 (56.9)		
	Gestational diabetes	8 (15.7)		
	IIOC	4 (7.8)		
	Preterm labor	4 (7.8)		
	Placenta previa	2 (3.9)		
	Preeclampsia (eclampsia)	1 (2.0)		
	Cervical polyp	1 (2.0)		
	Miscellaneous	2 (3.9)		
	No	102 (68.9)	22.95±7.04	
Illness before pregnancy	Yes	8 (5.4)	24.38±3.74	0.52 (.602)
	No	140 (94.6)	24.02±7.26	

IIOC: Incompetent internal os of cervix; KRW: Korean won (1 million is approximately 900 US dollars).

† Multiple responses,

‡ Scheffé test.

**Table 2.** Levels of main variables (N=148)

Variable	Mean±SD	Possible range	Data range	n (%)
Maternal knowledge	14.42±4.67	0–21	4–21	
Social support	45.88±7.81	12–60	28–60	
Pregnancy stress (cut-off: >17)	23.09±7.11	0–48	2–40	117 (79.1)
Negative affect (cut-off: >12)	19.94±6.10	0–33	2–31	127 (85.8)
Partner involvement (cut-off: >7)	3.16±2.90	0–15	0–15	12 (8.1)

**Table 3.** Correlations among pregnancy stress, pregnancy stress, maternal knowledge, and social support (N=148)

Variables	Pregnancy stress	Maternal knowledge
	r (*p*)	r (*p*)
Pregnancy stress	1	
Maternal knowledge	–.08 (.309)	1
Social support	–.37 (<.001)	.28 (.001)

**Table 4.** Factors influencing pregnancy stress (N=148)

Variable	B	SE	β	t (*p*)
(Constant)	36.71	3.43		10.70 (<.001)
Occupation^†^	–1.87	1.08	–.13	–1.74 (.085)
Relationship with in-laws^†^	–0.62	1.17	–.04	–0.53 (.595)
Marital satisfaction^†^	–3.48	1.64	–.18	–2.12 (.036)
Planned pregnancy^†^	–2.14	1.44	–.12	–1.49 (.138)
Gestational period (week)^†^				
1st trimester	0.77	1.21	.05	0.63 (.527)
3rd trimester	–2.97	1.75	–.13	–1.69 (.093)
Main source of pregnancy information^†^	–3.96	1.53	–.21	–2.59 (.011)
Maternal knowledge	0.08	0.12	.05	0.68 (.495)
Social support	–0.17	0.08	–.19	–2.10 (.038)
	R^2^=.274, adjusted R^2^=.227, F(*p*)=5.80 (<.001)			

† References were occupation (no); relationship with in-law (average to not amicable); marital satisfaction (moderate to dissatisfied); planned pregnancy (no); gestational period (2nd trimester); main source of pregnancy information (mass media).

## References

[1] Cutler A, McNamara B, Qasba N, Kennedy HP, Lundsberg L, Gariepy A (2018). “I just don’t know”: an exploration of women’s ambivalence about a new pregnancy. Women’s Health Issues.

[2] Staneva AA, Bogossian F, Wittkowski A (2015). The experience of psychological distress, depression, and anxiety during pregnancy: a meta-synthesis of qualitative research. Midwifery.

[3] http://kosis.kr/statHtml/statHtml.do?orgId=101&tblId=DT_1B8000H&checkFlag=N.

[4] Chung MR, Kim HM, Kang SK (2017). Assessment on the needs of pregnant women regarding pregnancy, childbirth, and parenting-related support policies. Korean J Early Childhood Educ.

[5] Alderdice F, Lynn F (2011). Factor structure of the prenatal distress questionnaire. Midwifery.

[6] Obrochta CA, Chambers C, Bandoli G (2020). Psychological distress in pregnancy and postpartum. Women Birth.

[7] Woods SM, Melville JL, Guo Y, Fan MY, Gavin A (2010). Psychosocial stress during pregnancy. Am J Obstet Gynecol.

[8] Yuksel F, Akin S, Durna Z (2014). Prenatal distress in Turkish pregnant women and factors associated with maternal prenatal distress. J Clin Nurs.

[9] Christian LM (2012). Physiological reactivity to psychological stress in human pregnancy: current knowledge and future directions. Prog Neurobiol.

[10] Lima SAM, El Dib RP, Rodrigues MR (2018). Is the risk of low birth weight or preterm labor greater when maternal stress is experienced during pregnancy? A systematic review and meta-analysis of cohort studies. PLoS One.

[11] Kwon MK, Bang KS (2011). Relationship of prenatal stress and depression to maternal-fetal attachment and fetal growth. J Korean Acad Nurs.

[12] Fatima M, Srivastav S, Mondal AC (2017). Prenatal stress and depression associated neuronal development in neonates. Int J Dev Neurosci.

[13] Hong HJ, Mun HJ (2011). The influences of stress and social support during pregnancy on maternal attachment in infancy: An examination of postpartum depression and its mediating effects. Korean J Human Develop.

[14] Fontein-Kuipers Y, Ausems M, Budé L, Van Limbeek E, De Vries R, Nieuwenhuijze M (2015). Factors influencing maternal distress among Dutch women with a healthy pregnancy. Women Birth.

[15] Engidaw NA, Mekonnen AG, Amogne FK (2019). Perceived stress and its associated factors among pregnant women in Bale zone Hospitals, Southeast Ethiopia: a cross-sectional study. BMC Res Notes.

[16] Hong K, Hwang H, Han H (2021). Perspectives on antenatal education associated with pregnancy outcomes: systematic review and meta-analysis. Women Birth.

[17] Sayakhot P, Carolan-Olah M (2016). Internet use by pregnant women seeking pregnancy-related information: a systematic review. BMC Pregnancy Childbirth.

[18] Racine N, Plamondon A, Hentges R, Tough S, Madigan S (2019). Dynamic and bidirectional associations between maternal stress, anxiety, and social support: the critical role of partner and family support. J Affect Disord.

[19] Jonsdottir SS, Thome M, Steingrimsdottir T (2017). Partner relationship, social support and perinatal distress among pregnant Icelandic women. Women Birth.

[20] Staneva A, Bogossian F, Pritchard M, Wittkowski A (2015). The effects of maternal depression, anxiety, and perceived stress during pregnancy on preterm birth: a systematic review. Women Birth.

[21] Barber CC, Steadman J (2018). Distress levels in pregnant and matched non-pregnant women. Aust N Z J Obstet Gynaecol.

[22] Vieten C, Laraia BA, Kristeller J (2018). The mindful moms training: development of a mindfulness-based intervention to reduce stress and overeating during pregnancy. BMC Pregnancy Childbirth.

[23] Solivan AE, Xiong X, Harville EW, Buekens P (2015). Measurement of perceived stress among pregnant women: a comparison of two different instruments. Matern Child Health J.

[24] Oh JS, Wright G, Coulam CB (2002). Gestational sac diameter in very early pregnancy as a predictor of fetal outcome. Ultrasound Obstet Gynecol.

[25] Cohen J (1988). Statistical power analysis for the behavioral sciences.

[26] Hwang RH (2019). Effects of state-anxiety and dyadic adjustment on pregnant women's pregnancy stress. J Digit Converg.

[27] Pop VJ, Pommer AM, Pop-Purceleanu M, Wijnen HA, Bergink V, Pouwer F (2011). Development of the Tilburg Pregnancy Distress Scale: the TPDS. BMC Pregnancy Childbirth.

[28] Beaton DE, Bombardier C, Guillemin F, Ferraz MB (2000). Guidelines for the process of cross-cultural adaptation of self-report measures. Spine (Phila Pa 1976).

[29] Kim HW (1998). Model construction of maternal identity in primigravida. J Korean Acad Nurs.

[30] Zimet GD, Dahlem NW, Zimet SG, Farley GK (1988). The multidimensional scale of perceived social support. J Pers Assess.

[31] Çapik A, Pasinlioglu T (2015). Validity and reliability study of the Tilburg Pregnancy Distress Scale into Turkish. J Psychiatr Ment Health Nurs.

[32] Hicyilmaz BD (2021). Characteristics associated with prenatal distress in Turkish women: a cross-sectional study. J Midwifery Reprod Health.

[33] Kuipers J, Henrichs J, Evans K (2020). A comparison of the Fear of Childbirth Scale with the Tilburg Pregnancy Distress Scale to identify childbirth-related fear in a sample of Dutch pregnant women: a diagnostic accuracy comparative cross-sectional study. Int J Nurs Stud.

[34] Boekhorst MG, Beerthuizen A, Van Son M, Bergink V, Pop VJ (2020). Psychometric aspects of the Tilburg Pregnancy Distress Scale: data from the HAPPY study. Arch Womens Ment Health.

[35] Cho HW, Woo JY (2013). The relational structure modeling between variables related with postpartum depression. Korean J Couns Psychother.

[36] Kim YK, Lim KH (2018). Risk factors for premature birth among premature obstetric labor women: a prospective cohort study. Korean J Women Health Nurs.

[37] Zamani P, Ziaie T, Lakeh NM, Leili EK (2019). The correlation between perceived social support and childbirth experience in pregnant women. Midwifery.

